# Digitalization of Health Care: Findings From Key Informant Interviews in Sweden on Technical, Regulatory, and Patient Safety Aspects

**DOI:** 10.2196/38746

**Published:** 2022-09-01

**Authors:** Björn Ekman, Hans Thulesius, Jens Wilkens, Eva Arvidsson

**Affiliations:** 1 Department of Clinical Sciences Lund University Malmö Sweden; 2 Futurum Region Jönköpings län Jönköping Sweden

**Keywords:** digitalization, regulation, patient safety, quality of care, interview, Sweden, health care, digital health, decision-making, digital care

## Introduction

Over the past few decades, digital technologies for health care have been introduced to improve effectiveness, reduce costs, and enhance access [1]. The case for digital health care increased dramatically during the COVID-19 pandemic [2,3]. However, less is known about some of the broader conditions under which these technologies would be able to contribute to improving health care [4]. To inform policy development from a national perspective, we conducted key informant interviews around 3 domains of digitalization: (1) infrastructure and skills, (2) regulatory considerations, and (3) patient safety and quality of care (see, for example, Desveaux et al [4]).

## Methods

### Overview

In spring 2021, we interviewed 24 representatives identified in a scoping exercise across 4 areas: providers of health services, researchers active in the field, regional health authorities, and national-level organizations. The responses were analyzed using conventional content analysis [5].

### Ethics Approval

The Swedish Ethical Review Authority reviewed the study (2020-04381; September 29, 2020).

## Results

### Infrastructure and Skills

The technical conditions for health care digitalization in Sweden are favorable. Broadband is generally available; most people have access to a computer, tablet, or smartphone; and the level of acceptability of using such tools is high. The existence of a national digital system for secure identification was also noted.

However, challenges and concerns were also noted, including variation in access and ability to navigate digital tools across population groups. Older adults, immigrants, and persons living with a disability may have difficulties accessing services by means of digital tools. The COVID-19 pandemic has accelerated the application of digital primary care in Sweden [6].

### Regulatory Aspects

Respondents noted the largely weak regulatory environment for health care in Sweden, including the absence of national policies for digital care and a host of regulators with unclear mandates and roles.

The lack of a strategic approach at the national level and the strong decentralization and autonomy of the 21 Swedish health care regions lead to significant local variation in the approach to digitalization in health care. Examples included a plethora of digital platforms for health services, different requirements with respect to public purchasing of services, and variations in the application of the need for data sharing and storage. A particular concern was the case-based payment for digital care while traditional primary care services are reimbursed through capitation.

### Patient Safety and Quality-of-Care Issues

Respondents noted both opportunities and risks with respect to patient safety and quality of care. On the one hand, digital technologies were seen as safe and may support improved quality of care as more data are available for clinical decision-making. On the other hand, there may be limitations to telemedicine for certain conditions and for patients with heavier demands, suggesting a further need to evaluate the effects. The risk of data mismanagement when using digital models of health care was also noted.

However, respondents also noted that there are similar risks with existing in-person models of care and that it is partly a question of finding the right balance between using digital technologies in a way that maximizes their effectiveness while managing the risks of such technologies. During the COVID-19 pandemic, digital care proved to be the safest option.

## Discussion

### Principal Findings

The continued integration of advanced digital technologies in health care is likely to transform much of medical service delivery, both in scope and content. Understanding the implications across the technical, regulatory, and patient safety aspects will be critical for the successful digitalization of health care.

The findings suggest that ensuring the technical conditions for digital care may be challenging even in a relatively advanced country such as Sweden. In resource-limited countries, this may be even more so [7]. The COVID-19 pandemic may have exposed some of these issues [6].

Failure to ensure a clear, effective, and transparent regulatory environment for digital care may risk jeopardizing the realization of the opportunities that digital technologies present and aggravating the risks that such technologies also entail. These factors relate critically to patient safety and to the quality of care. Ongoing analysis of the material using grounded theory will extend the analysis and deepen the understanding of these issues.

### Conclusion

The successful application of digital technologies for health care will depend on the strategic management of a set of technical, regulatory, and patient safety aspects. Even in advanced countries, there may be concerns related to each of these. Policy makers and regulatory agencies need to be made aware of these challenges. Further research will be needed to advance the understanding of the digitalization of health care along these dimensions.
